# Rare and varied presentations of plasmablastic lymphoma: A case series

**DOI:** 10.1177/2050313X251408241

**Published:** 2026-01-12

**Authors:** Abdulrahman F. Al-Mashdali, Farah Jibril, Syed M. Rizvi, Ruba Taha, Mohammad Bakr, Sarah A. Elkourashy

**Affiliations:** 1 Department of Hematology and Bone Marrow Transplant, National Center for Cancer Care and Research (NCCCR), Hamad Medical Corporation, Doha, Qatar; 2 Department of Pharmacy, National Center for Cancer Care and Research (NCCCR), Hamad Medical Corporation, Doha, Qatar; 3 Department of Pathology, Hamad Medical Corporation, Doha, Qatar; 4 Weill Cornell Medicine – Qatar (WCM-Q), Doha, Qatar

**Keywords:** plasmablastic lymphoma, extranodal lymphoma, bortezomib-EPOCH, treatment outcomes, prognostic factors

## Abstract

Plasmablastic lymphoma is a rare and aggressive variant of diffuse large B-cell lymphoma characterized by heterogeneous clinical presentations and poor outcomes. This study presents a comprehensive review integrated with three cases demonstrating diverse clinical manifestations and treatment outcomes. We conducted a detailed analysis of three plasmablastic lymphoma cases diagnosed and treated at our institution between 2022 and 2023, incorporating clinical presentations, diagnostic findings, treatment approaches, and outcomes. These cases were analyzed in the context of current literature and treatment guidelines. The cases included an HIV-positive male with perianal plasmablastic lymphoma, an HIV-negative female with gastric plasmablastic lymphoma arising from marginal zone lymphoma, and an HIV-negative female with retroperitoneal plasmablastic lymphoma. Two patients achieved complete remission with bortezomib plus dose-adjusted-etoposide, prednisolone, vincristine, cyclophosphamide, and doxorubicin therapy, while one experienced treatment failure and death. Epstein–Barr virus positivity was observed in two cases. Treatment-related complications included peripheral neuropathy and organ failure. The cases demonstrated variable outcomes independent of HIV status but correlating with age and performance status. Our series highlights the diverse presentation patterns of plasmablastic lymphoma and validates known prognostic factors while demonstrating the efficacy of contemporary treatment approaches. The outcomes underscore the importance of individualized therapy and careful patient selection for intensive treatment regimens.

## Introduction

Plasmablastic lymphoma (PBL) is a distinct entity classified by the World Health Organization as an aggressive CD20-negative non-Hodgkin lymphoma.^1^ While predominantly associated with HIV infection, accounting for ~2% of HIV-related lymphomas, PBL has been increasingly recognized in other clinical settings. According to the National Cancer Database, PBL represented 0.7% of all diffuse large B-cell lymphoma (DLBCL) cases between 2010 and 2013, with 481 documented cases.^2^

The spectrum of PBL extends beyond HIV-associated cases, occurring in various contexts of immunodeficiency, including post-solid organ transplantation, autoimmune conditions, and transformation from indolent lymphoproliferative disorders. Rare cases have also been reported in immunocompetent individuals. The disease typically follows an aggressive clinical course, characterized by poor response to conventional chemotherapy and dismal survival outcomes.^3,4^

PBL presents significant diagnostic challenges due to its heterogeneous clinical manifestations and complex histological and immunohistochemical features. In this case series, we present three patients with PBL from Qatar, contributing to the growing body of knowledge about this rare and aggressive lymphoma, with particular emphasis on its clinical presentation, diagnostic approach, and treatment outcomes in our regional context.

## Case 1

A 44-year-old Pakistani male with advanced HIV infection, diagnosed in May 2022, presented to the emergency department with a 10-day history of anal pain. The patient had a history of poor compliance with his antiretroviral therapy (ART) and had been off treatment since December 2022 due to financial reasons, including a period of travel to Pakistan. At the time of his HIV diagnosis in May 2022, his baseline studies showed profound immunosuppression with a CD4 count of 69 cells/µL (8.7%) and a high HIV viral load of 287,000 copies/mL. Genotypic resistance testing revealed an E138A mutation, associated with low-level resistance to rilpivirine, but no other resistance to his eventual regimen.

Initial evaluation included a computerized tomography (CT) scan that revealed a 5 × 7 cm soft tissue lesion in the perianal region. The patient was initially admitted to the Acute Care Surgery team with a presumptive diagnosis of perianal abscess and underwent incision and drainage of a perianal fistula.

Following the initial surgical intervention, the patient’s clinical course was marked by multiple emergency department presentations (three additional visits) due to persistent perianal pain and rapidly progressive anal swelling. Physical examination during these visits revealed a large 5 × 6 cm fungating, tender, gangrenous anal mass characterized by a foul smell and purulent discharge, with an infected suture noted on the mass. The patient reported occasional stool incontinence, occurring in small amounts without straining.

Laboratory investigations revealed several significant findings. While white blood cell count, platelets, and neutrophils were within normal limits, the patient demonstrated marked anemia with a hemoglobin of 6.5 g/dL. Other notable laboratory values included elevated lactate dehydrogenase (294 U/L). A comprehensive infectious disease workup revealed positive HIV and Epstein–Barr virus (EBV) status (including EBV PCR), while hepatitis B and C serologies were negative.

The diagnostic workup included a pathology review, which confirmed the diagnosis of PBL. A bone marrow biopsy showed hypercellularity (70%–85%) but notably revealed no evidence of lymphoma involvement. PET/CT imaging demonstrated an intensely hypermetabolic anorectal mass corresponding to the biopsy-proven lymphoma, along with an intensely hypermetabolic perirectal lymph node and innumerable osseous foci suspicious for lymphomatous infiltration, leading to a final staging of Stage IV disease.

Following discussion in a multidisciplinary team meeting, the patient was initiated on a treatment regimen consisting of bortezomib combined with dose-adjusted EPOCH (etoposide, prednisolone, vincristine, cyclophosphamide, and doxorubicin) chemotherapy. Simultaneously, ART was re-initiated on June 13, 2022, with the regimen Bictegravir/Emtricitabine/Tenofovir alafenamide. The treatment course was complicated by several significant adverse events, including febrile neutropenia and *Clostridioides difficile* colitis.

The patient demonstrated a significant virological and immunological response to ART alongside his chemotherapy. After ~3 months on Biktarvy, a repeat test in September 2022 showed a dramatic decline in the HIV viral load to 317 copies/mL and an increase in the CD4 count to 145 cells/µL. The interim PET/CT scan following four cycles of chemotherapy demonstrated a very good response with near-complete remission. The patient successfully completed all six planned cycles of chemotherapy, achieving complete remission. By the end of chemotherapy, subsequent monitoring showed further improvement, with the HIV viral load suppressed to <50 copies/mL and a CD4 count that had risen to 260 cells/µL.

Although the patient was offered hematopoietic stem cell transplantation as consolidation therapy, he declined this option and elected to return to his home country, resulting in loss to follow-up.

## Case 2

A 45-year-old Filipino female with no significant past medical history presented with jaundice, abdominal pain, fever, and dizziness. The patient reported months of fatigue but denied constitutional symptoms such as weight loss or night sweats.

Initial laboratory investigations revealed severe anemia (hemoglobin 6.0 g/dL) requiring blood transfusion, while other blood counts were normal. Liver function tests were notably deranged with elevated alkaline phosphatase (562 U/L), alanine aminotransferase (ALT; 97 U/L) and aspartate aminotransferase (AST; 92 U/L), and total bilirubin (56 μmol/L). Serum protein electrophoresis showed low albumin with polyclonal gammopathy, suggesting chronic inflammation. Notably, HIV testing was negative.

**Figure 1.**
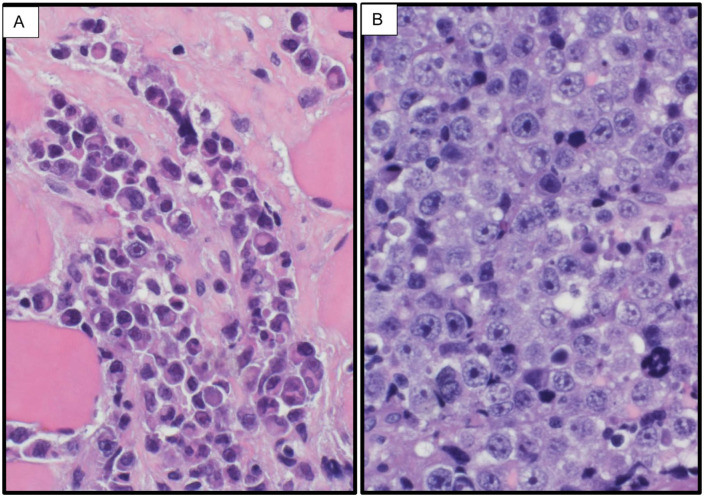
Histopathological spectrum of the neoplasm in two representative cases (H&E stain). (a) *Case 3*: The neoplasm shows a range of maturation, from plasma cells with a small eccentric nucleus and eosinophilic cytoplasm featuring a perinuclear halo, to frank plasmablasts with prominent central nucleoli and high nuclear-to-cytoplasmic ratios. (b) *Case 1*: The neoplasm is composed predominantly of typical plasmablasts, with few immature forms and an absence of well-differentiated plasma cells.

**Figure 2.**
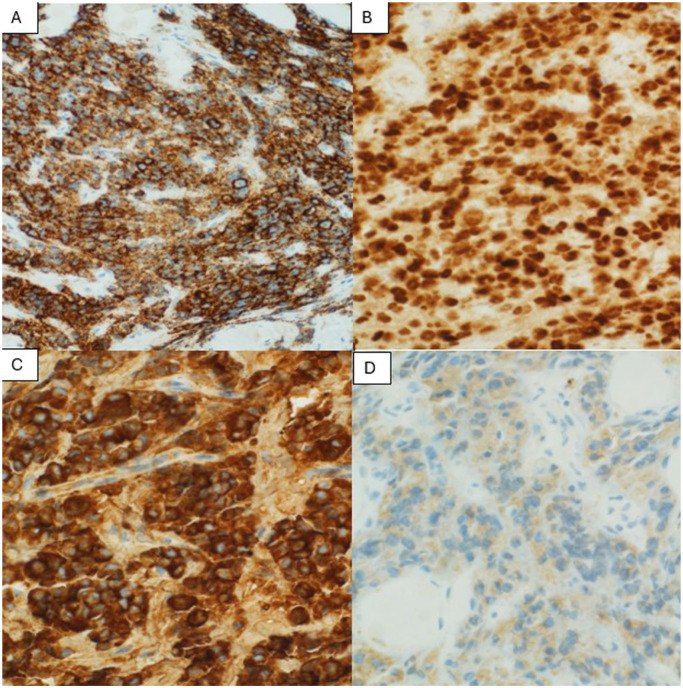
Immunohistochemical profile of the neoplastic cells. (a) Strong membranous CD138 positivity highlights the neoplastic plasma cell population. All cases were negative for CD20 (not shown), confirming the absence of a B-cell phenotype. (b) Nuclear expression of the plasma cell transcription factor MUM1/IRF4. (c) Negative kappa light chain staining. (d) Intense cytoplasmic lambda light chain expression, indicating a monotypic plasma cell population. The kappa-negative (c) and lambda-positive (d) staining pattern confirms light chain restriction.

**Figure 3.**
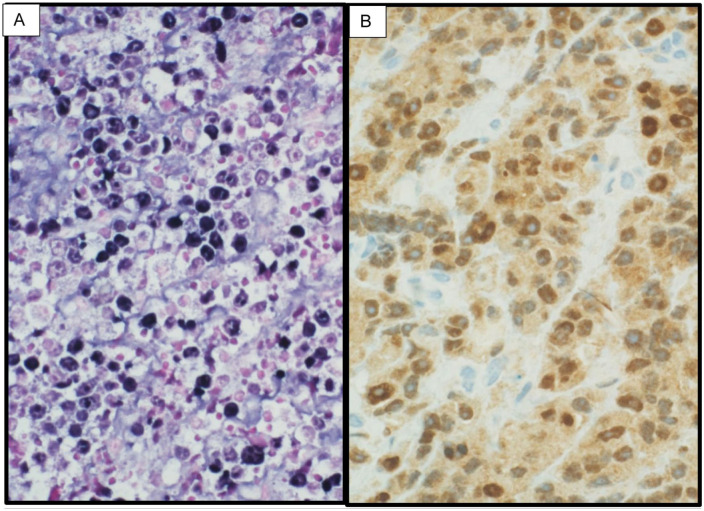
In-situ hybridization and immunohistochemical findings. (a) *Case 1*: EBER in-situ hybridization shows strong nuclear positivity. (b) *Case 3*: Immunohistochemistry for MYC shows strong nuclear protein expression in the neoplastic plasmablasts. EBER: Epstein–Barr virus-encoded RNA.

**Figure 4.**
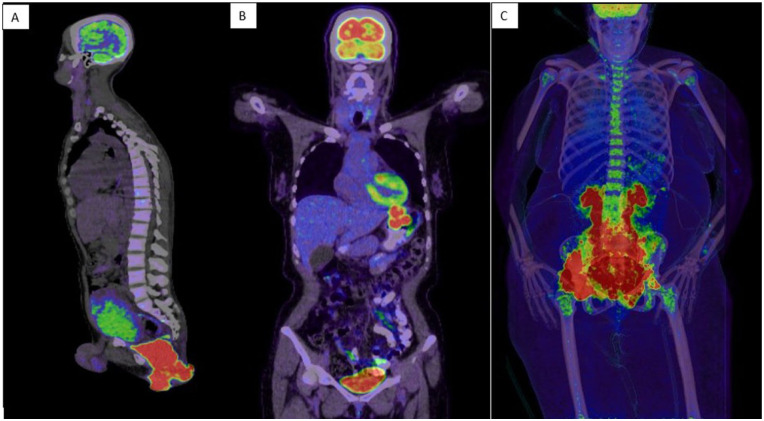
(a) FDG PET/CT imaging revealed an intensely hypermetabolic anorectal mass corresponding to the biopsy-proven lymphoma, accompanied by an intensely hypermetabolic perirectal lymph node. The scan also demonstrated innumerable osseous foci suspicious for lymphomatous infiltration, resulting in Stage IV disease classification. (b) FDG PET/CT staging demonstrated Stages II–E disease with isolated gastric involvement. (c) FDG PET/CT showed a large retroperitoneal enhancing mass extending throughout the pelvic cavity and lower abdomen, with extension from the ischiorectal fossa to the right gluteus muscles. This mass caused significant compression of surrounding structures, notably including bilateral ureteral encasement with resultant hydroureteronephrosis. CT: computerized tomography.

Upper endoscopy revealed multiple polypoidal masses along the stomach’s greater and lesser curvatures, with the largest measuring 3 × 3 cm, showing bilobular morphology with irregular, friable, and ulcerated surface. Histopathological examination confirmed PBL positive for EBV and CD138, while negative for CD20 and CD79a, arising in a background of marginal zone lymphoma (Figures 1–3). PET/CT staging revealed Stages II–E disease, with no bone marrow involvement (Figure 4(b)).

The patient was treated with DA-EPOCH plus bortezomib. The patient’s treatment course required judicious modifications to manage a known, cumulative toxicity. The development of grade 2 peripheral neuropathy necessitated a vincristine dose reduction in cycle 3 and the subsequent omission of bortezomib after cycle 4 to prevent permanent neurological damage, adhering to standard toxicity management protocols. Despite this modification, the patient achieved a complete metabolic response on the interim PET scan, which was maintained after completing six cycles of DA-EPOCH and four cycles of bortezomib. At the time of this report, the patient remains in complete remission with regular follow-up.

## Case 3

A 62-year-old Nigerian female presented to the Emergency Department in August 2023 with bilateral leg edema, generalized weakness, shortness of breath, and urinary symptoms. Her medical history was significant for chronic kidney disease, hypertensive heart disease, cardiomyopathy with a reduced ejection fraction (left ventricular ejection fraction of 37%), and a recently diagnosed rectal malignancy. On presentation, the patient was severely ill with an ECOG performance status of 4. Initial laboratory studies revealed significant abnormalities, including anemia (hemoglobin 8.0 g/dL), severe hyponatremia (sodium 108 mmol/L), and markedly elevated creatinine (849 μmol/L). Total protein was elevated at 102 g/L with albumin of 34 g/L, and β2-microglobulin was 15.30 mg/L.

FDG PET/CT revealed a large retroperitoneal enhancing mass occupying the pelvic cavity and lower abdomen, extending from the ischiorectal fossa to the right gluteus muscles, causing significant compression of surrounding structures, including bilateral ureteral encasement with hydroureteronephrosis (Figure 4(c)). Bone-destructive lesions were noted in the right ilium and sacrum, with evidence of a pathological fracture in the right iliac bone. A biopsy of the retroperitoneal mass confirmed a plasma cell neoplasm, consistent with PBL (EBV-encoded RNA (EBER) negative; Figures 1–3). Bone marrow examination revealed involvement by PBL, confirming disseminated disease.

The patient’s multiple comorbidities profoundly limited and complicated the therapeutic strategy from the outset. Her baseline chronic kidney disease was exacerbated by PBL-induced bilateral ureteral obstruction, leading to end-stage renal failure requiring hemodialysis. This rendered several chemotherapeutic agents prohibitively toxic and necessitated careful dose adjustment. Furthermore, her preexisting cardiomyopathy (LVEF 37%) contraindicated the use of full-dose anthracyclines, a cornerstone of aggressive lymphoma regimens, due to the high risk of irreversible heart failure. This directly led to the initial choice of an attenuated “mini-CHOP” (cyclophosphamide, doxorubicin, vincristine, and prednisone) regimen, inherently compromising anti-tumor efficacy from the start. Her critical ECOG performance status of four further signaled an inability to tolerate aggressive, curative-intent chemotherapy, forcing the medical team to balance disease control with the high risk of treatment-related mortality.

The hospital course was complicated by end-stage renal failure requiring hemodialysis and bilateral percutaneous nephrostomy placement. The patient developed Extended Spectrum Beta Lactmase (ESBL) bacteremia requiring ICU admission and experienced sepsis-induced atrial fibrillation. The aggressive biology of the PBL, operating within this compromised host, created a vicious cycle of complications that ultimately led to treatment resistance and death. The massive, obstructive retroperitoneal mass was the primary driver of morbidity, directly causing renal failure. The initial, attenuated CEOP chemotherapy (cyclophosphamide, etoposide, vincristine, and prednisone), mandated by her cardiac and renal status, was insufficient to control the disease and was complicated by respiratory failure requiring intubation.

However, despite this aggressive therapy, follow-up PET/CT showed no significant improvement in the disease burden. The patient’s condition deteriorated with severe pancytopenia requiring continuous blood product support. She ultimately developed gross hemoptysis and septic shock, requiring re-intubation. Despite maximal supportive care and vasopressor support, the patient could not be resuscitated and was declared dead.

## Discussion

PBL represents a significant diagnostic challenge due to its rarity and heterogeneous presentation patterns across different ethnic and geographic populations. The limited reported cases have created substantial knowledge gaps in understanding its clinical behavior and natural history. A distinguishing feature is its predilection for extranodal involvement (53% vs 35% in DLBCL), despite comparable rates of advanced-stage disease (57% and 55%, respectively). While PBL can potentially involve any organ system, it demonstrates a characteristic pattern of extranodal distribution, with the oral cavity representing a primary site of involvement, followed by the gastrointestinal tract, lymph nodes, paranasal sinuses, and cutaneous manifestations. Less frequent sites include osseous structures, genitourinary system, central nervous system, hepatic tissue, and pulmonary parenchyma.^2,5,6^ Our case series exemplifies this heterogeneity, with presentations ranging from perianal involvement in an HIV-positive male (Case 1), to gastric involvement in an HIV-negative female (Case 2), and a rare retroperitoneal presentation with extensive local invasion in another HIV-negative female (Case 3).^7^

Definitive diagnosis requires comprehensive tissue evaluation through excisional, incisional, or core needle biopsies, as fine-needle aspiration alone is insufficient due to PBL’s distinctive morphology and characteristic absence of CD20 expression.^4^ The immunophenotypic profile is distinctive and crucial for diagnosis, characterized by strong expression of plasma cell markers (CD79a, IRF-4/MUM-1, BLIMP-1, CD38, CD138), negative expression of typical B-cell markers (CD20, PAX-5), variable expression of CD19 and CD45, and high proliferation index with near-universal Ki-67 positivity. Some cases may show aberrant expression of T-cell markers (CD2, CD3, CD4, or CD8), while CD10, BCL2, and BCL6 expression is infrequent, consistent with a nongerminal center origin.^8,9^ Our cases demonstrated these characteristic features, with Case 2 being particularly noteworthy for its background of marginal zone lymphoma, adding to the complexity of diagnosis. EBV involvement, typically present in 66% of cases and detected through EBER or EBV LMP-1, was observed in Cases 1 and 2 but absent in Case 3. Molecular testing often reveals MYC rearrangements in two-thirds of cases, though all our patients were negative for MYC rearrangements. Disease staging utilized PET/CT imaging following the Lugano modification of the Ann Arbor staging system, which proved crucial for initial assessment and response monitoring.^6,10^

Several clinical parameters influence prognosis, including advanced age (⩾60 years), disseminated disease at presentation, and elevated International Prognostic Index scores (high or high-intermediate), which consistently correlate with inferior survival outcomes. The prognostic significance of biological and molecular features, including HIV status, EBV expression, and MYC gene rearrangements, remains controversial and requires further investigation through larger, prospective studies.^4^ Our cases validate these prognostic factors, with Case 3 demonstrating poor outcomes associated with advanced age and multiple comorbidities, while Case 1 showed a good response despite HIV positivity and advanced disease. Cases 1 and 2 achieved complete remission despite different HIV statuses, supporting the complex relationship between HIV status and outcomes.

PBL typically follows an aggressive and relapsing clinical course, which results in poor outcomes. While CHOP was historically standard, Current National Comprehensive Cancer Network (NCCN) guidelines recommend more intensive regimens such as CODOX-M/IVAC (cyclophosphamide, vincristine, doxorubicin, high-dose methotrexate / ifosfamide, etoposide, and cytarabine), EPOCH (etoposide, prednisone, vincristine, cyclophosphamide, and doxorubicin), and hyper-CVAD (hyperfractionated cyclophosphamide, vincristine, doxorubicin, and dexamethasone).^4,5^ Recent advances include high-dose chemotherapy with novel agents like bortezomib and lenalidomide, emerging therapies such as immune checkpoint inhibitors and CAR-T therapy, and consideration of autologous stem cell transplantation in eligible patients, especially after the first complete remission or in relapsed cases.^4^ Our cases demonstrate modern treatment approaches, with all patients receiving bortezomib combined with DA-EPOCH. Outcomes varied significantly: Case 1 achieved complete remission but declined transplant and was lost to follow-up, Case 2 demonstrated sustained complete remission with successful management of treatment-related complications, and Case 3 showed treatment resistance with rapid clinical deterioration and a fatal outcome.

Treatment complications were significant across our series, highlighting the challenges of delivering intensive therapy. Case 2 developed peripheral neuropathy requiring dose modifications of vincristine and bortezomib but successfully completed therapy with appropriate supportive care. Case 3 experienced multiple organ failure, sepsis, and ultimately a fatal outcome despite aggressive supportive measures. Case 1 completed therapy despite complications but declined further consolidation treatment, emphasizing the importance of patient preferences and social factors in treatment decisions. This comprehensive review, enriched by our case series, highlights the complex nature of PBL and the importance of individualized treatment approaches considering patient factors such as age, comorbidities, performance status, and ability to tolerate therapy. The varied outcomes underscore the need for careful patient selection for intensive therapy and the critical role of supportive care during treatment. The integration of novel therapies and the potential role of consolidative strategies, particularly in responding patients, remain areas of active investigation in this challenging disease.

## Conclusion

PBL demonstrates remarkable heterogeneity in clinical presentation, as evidenced by our cases ranging from perianal to gastric and retroperitoneal involvement. The integration of bortezomib with DA-EPOCH represents a promising therapeutic approach, though outcomes remain variable and heavily influenced by patient characteristics. Our series validates established prognostic factors, particularly highlighting the impact of age and performance status over HIV status in determining outcomes. Treatment-related complications necessitate careful patient selection and robust supportive care measures during intensive therapy.

## References

[1] Alaggio R Amador C Anagnostopoulos I , et al. The 5th edition of the World Health Organization classification of haematolymphoid tumours: lymphoid neoplasms. Leukemia 2022; 36: 1720–1748. 35732829 10.1038/s41375-022-01620-2 PMC9214472

[2] Qunaj L Castillo JJ Olszewski AJ. Survival of patients with CD20-negative variants of large B-cell lymphoma: an analysis of the National Cancer Data Base. Leuk Lymphoma 2018; 59: 1375–1383. 29019447 10.1080/10428194.2017.1387912

[3] Teruya-Feldstein J Chiao E Filippa DA , et al. CD20-negative large-cell lymphoma with plasmablastic features: a clinically heterogenous spectrum in both HIV-positive and -negative patients. Ann Oncol 2004; 15: 1673–1679. 15520070 10.1093/annonc/mdh399

[4] Ramirez-Gamero A Martínez-Cordero H Beltrán BE , et al. Plasmablastic lymphoma: 2024 update on diagnosis, risk stratification, and management. Am J Hematol 2024; 99: 1586–1594. 38767403 10.1002/ajh.27376

[5] Castillo JJ Bibas M Miranda RN. The biology and treatment of plasmablastic lymphoma. Blood 2015; 125: 2323–2330. 25636338 10.1182/blood-2014-10-567479

[6] Castillo J Pantanowitz L Dezube BJ. HIV-associated plasmablastic lymphoma: lessons learned from 112 published cases. Am J Hematol 2008; 83: 804–809. 18756521 10.1002/ajh.21250

[7] Takahashi Y Saiga I Fukushima J-I , et al. Plasmablastic lymphoma of the retroperitoneum in an HIV-negative patient. Pathol Int 2009; 59: 868–873. 20021612 10.1111/j.1440-1827.2009.02457.x

[8] Vega F Chang C-C Medeiros LJ , et al. Plasmablastic lymphomas and plasmablastic plasma cell myelomas have nearly identical immunophenotypic profiles. Mod Pathol 2005; 18: 806–815. 15578069 10.1038/modpathol.3800355

[9] Morscio J Dierickx D Nijs J , et al. Clinicopathologic comparison of plasmablastic lymphoma in HIV-positive, immunocompetent, and posttransplant patients: single-center series of 25 cases and meta-analysis of 277 reported cases. Am J Surg Pathol 2014; 38: 875–886. 24832164 10.1097/PAS.0000000000000234

[10] Valera A Balagué O Colomo L , et al. IG/MYC rearrangements are the main cytogenetic alteration in plasmablastic lymphomas. Am J Surg Pathol 2010; 34: 1686–1694. 20962620 10.1097/PAS.0b013e3181f3e29f PMC2982261

